# Phosphorylation-Dependent Inhibition of Akt1

**DOI:** 10.3390/genes9090450

**Published:** 2018-09-07

**Authors:** Nileeka Balasuriya, McShane McKenna, Xuguang Liu, Shawn S. C. Li, Patrick O’Donoghue

**Affiliations:** 1 Department of Biochemistry, Schulich School of Medicine and Dentistry, The University of Western Ontario, London, ON N6A 5C1, Canada; bbalasur@uwo.ca (N.B.); mmcken22@uwo.ca (M.M.); xliu329@uwo.ca (X.L.); sli@uwo.ca (S.S.C.L.); 2 Department of Chemistry, Faculty of Science, The University of Western Ontario, London, ON N6A 5C1, Canada

**Keywords:** genetic code expansion, protein kinase B, phosphoinositide dependent kinase 1, phosphoseryl-tRNA synthetase, tRNA^Sep^

## Abstract

Protein kinase B (Akt1) is a proto-oncogene that is overactive in most cancers. Akt1 activation requires phosphorylation at Thr308; phosphorylation at Ser473 further enhances catalytic activity. Akt1 activity is also regulated via interactions between the kinase domain and the N-terminal auto-inhibitory pleckstrin homology (PH) domain. As it was previously difficult to produce Akt1 in site-specific phosphorylated forms, the contribution of each activating phosphorylation site to auto-inhibition was unknown. Using a combination of genetic code expansion and in vivo enzymatic phosphorylation, we produced Akt1 variants containing programmed phosphorylation to probe the interplay between Akt1 phosphorylation status and the auto-inhibitory function of the PH domain. Deletion of the PH domain increased the enzyme activity for all three phosphorylated Akt1 variants. For the doubly phosphorylated enzyme, deletion of the PH domain relieved auto-inhibition by 295-fold. We next found that phosphorylation at Ser473 provided resistance to chemical inhibition by Akti-1/2 inhibitor VIII. The Akti-1/2 inhibitor was most effective against pAkt1^T308^ and showed four-fold decreased potency with Akt1 variants phosphorylated at Ser473. The data highlight the need to design more potent Akt1 inhibitors that are effective against the doubly phosphorylated and most pathogenic form of Akt1.

## 1. Introduction

Protein kinase B (Akt) is a human serine–threonine kinase and a member of the AGC family of protein kinases [1,2]. The pathway regulated by Akt is the most commonly activated signaling pathway in human cancers [3]. Given that more than 50% of human tumors contain hyperactivated Akt [2], effective inhibition of active Akt has the potential to treat several distinct cancers. There are three *AKT* genes in humans, encoding the isozymes Akt1, Akt2, and Akt3. The Akt1 isozyme has well-established roles in many human cancers. Overactive Akt1 is a hallmark of diverse human malignancies [3,4] and linked to reduced survival outcomes [5,6]. Indeed, Akt1 is as a leading drug target in cancer [7,8]. Over 300 clinical trials have been completed or are under way that involve targeting the Akt1 signaling pathway [9,10].

**Figure 1:**
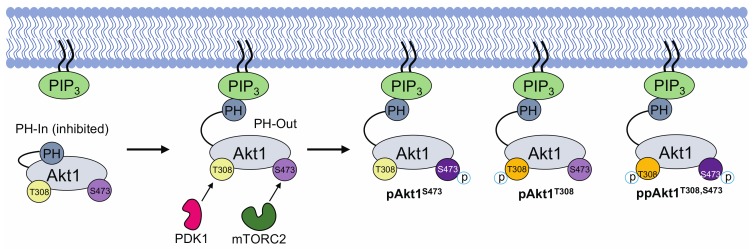
Simplified schematic of protein kinase B (Akt1) activation via phosphorylation of sites Thr308 and Ser473. The transition from Akt1’s inactive state (PH-in) to its fully active state (ppAkt1^T308/S473^) requires the release of pleckstrin homology (PH) domain–mediated auto-inhibition. This release occurs when Akt1’s PH domain interacts with PIP_3_ (PH-out). In the PH-out conformation, Akt1 is more susceptible to phosphorylation at Thr308 and Ser473 by phosphoinositide dependent kinase 1 (PDK1) and mechanistic target of rapamycin complex 2 (mTORC2), respectively. Upon release from PIP_3_, Akt1 distributes rapidly in the cytosol and translocates to the nucleus to phosphorylate >100 cellular proteins [2,17].

Akt1 is a key regulator of the phosphoinositide 3 kinase (PI3K)/Akt1 signaling cascade that controls cell growth and survival [1]. In human cells, the activation of Akt1 occurs in response to growth factor stimulation. Following activation by a receptor tyrosine kinase at the plasma membrane, PI3K phosphorylates its immediate downstream target, a lipid second messenger called phosphatidylinositol-4,5-bisphosphate (PIP_2_), converting PIP_2_ into phosphatidylinositol-3,4,5-triphosphate (PIP_3_) (Figure 1) [2]. Membrane-anchored PIP_3_ is a binding site for pleckstrin homology (PH) domain–containing proteins such as Akt1 and one of the upstream kinases that activates Akt1, phosphoinositide-dependent kinase 1 (PDK1) [11]. Co-localization of Akt1 with PDK1 leads to partial activation of Akt1 by PDK1-mediated phosphorylation of Thr308 in the kinase domain of Akt1. Mechanistic target of rapamycin complex 2 (mTORC2) is responsible for phosphorylating Akt1 at Ser473 in the C-terminal hydrophobic motif of Akt1 (Figure 1). The phosphorylation of Ser473 further increases the kinase activity of Akt1. Although the cellular role of Ser473 phosphorylation is not well defined, several studies point to the idea that pSer473 may impact Akt1 substrate selectivity (reviewed in [2]).

**Figure 2:**
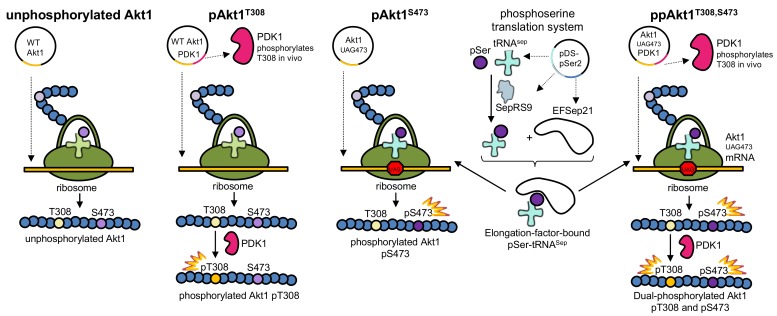
Production of Akt1 variants with programmed phosphorylation. To produce pAkt1^T308^, PDK1 (Akt1’s natural upstream kinase) was co-expressed along with Akt1. To produce pAkt1^S473^, the phosphoserine orthogonal translation system was used to genetically incorporate phosphoserine at position 473 in response to an amber (UAG) codon. The ppAkt1^T308,S473^ variant was produced by combining both methods. WT: wild type.

Leveraging our ability to produce Akt1 protein in specifically phosphorylated forms (Figure 2), we recently quantified the precise contribution of pThr308 and pSer473 to Akt1 activity in vitro and in mammalian cells [12]. In studies with purified full-length Akt1, we found that pAkt1^T308^ achieves 30% of the activity of the doubly phosphorylated kinase. We also observed in COS-7 cells that pSer473 is dispensable for Akt1 signaling [12]. In close agreement with our findings, studies in adipocytes found that half-maximal Akt1 cellular signaling activity is achieved when the kinase is 5–22% phosphorylated [13]. In studies of human tumors [12,13,14,15] and in clinical diagnostic settings [14,15], the phosphorylation status of Akt1 at Ser473 only is often used as a marker or proxy for Akt1 activity. Our work [12] and work by others [16] found that phosphorylation of Thr308 alone was sufficient for maximal Akt1 signal prorogation in cells. These results indicated that compared to Ser473, Thr308 phosphorylation status is a superior biomarker for Akt1 activity.

The N-terminal PH domain is auto-inhibitory to Akt1 activity. Due to previous roadblocks in preparing Akt1 with programmed phosphorylation(s), there are no reports that measure the contribution of a phosphate at each key regulatory site to the auto-inhibition of Akt1 by the PH domain. In the current model, Akt1 exists in an auto-inhibited (PH-in) and activated (PH-out) conformation. In its auto-inhibited conformation (PH-in), the PH domain binds between the N- and C-terminal lobes of the unphosphorylated Akt1 kinase domain. During growth factor–mediated activation of Akt1, the PH domain forms a new interaction with PIP_3_, causing it to move outward, away from the now more accessible kinase domain. This PH-out conformation is readily activated via phosphorylation at Thr308 by PDK1 and Ser473 by mTORC2. Disruption of the PH and kinase domain interaction was identified as a plausible cause of increased Akt1 phosphorylation and subsequent activity in cancer [18]. 

Here we quantified the ability of the PH domain to auto-inhibit Akt1 variants phosphorylated at either or both key regulatory sites Thr308 and Ser473. We produced novel recombinant Akt1 variants lacking the PH domain (ΔPH-Akt1) that also contained programmed phosphorylation at each site separately or with both sites phosphorylated. In the context of full-length phosphorylated Akt1 variants, we quantified the contribution of both regulatory phosphorylation sites to chemical inhibition with the PH domain–dependent allosteric inhibitor Akti-1/2.

## 2. Materials and Methods

### 2.1. Bacterial Strains and Plasmids

We designed a codon-optimized PH domain–deficient (ΔPH) human *AKT1* gene (residues 109–480), which was synthesized by ATUM (Newark, CA, USA). The ΔPH-*AKT1* gene was subcloned (*Nco*I/*Not*I) into an isopropyl β-D-1-thiogalactopyranoside (IPTG)–inducible T7*lac* promoter–driven pCDF-Duet1 vector with CloDF13-derived CDF replicon and streptomycin/spectinomycin resistance (pCDF-Duet1-ΔPHAkt1). The *PDPK1* gene was purchased from the Harvard PlasmidID repository service (plasmid ID: HsCD00001584; Boston, MA, USA) and subcloned (*KpnI/NdeI*) into the second multicloning site (MCS) of pCDF-Duet-1. Full-length pAkt1 variants were produced from pCDF-Duet1 plasmids as described previously [12]. The genetic code expansion system for phosphoserine (pSer) is encoded on the pDS-pSer2 plasmid [12,19,20], which contains 5 copies of tRNA^Sep^ [21], phosphoseryl-tRNA synthetase (SepRS9), and elongation factor Tu mutant (EFSep21) [22]. Incorporation of pSer also required site-directed mutagenesis of the Ser473 codon to TAG in the ΔPH-*akt1* constructs. Successful cloning was verified by DNA sequencing at the London Regional Genomics Centre (London, ON, Canada) and Genewiz (Cambridge, MA, USA). 

### 2.2. Protein and Phosphoprotein Production 

Recombinant Akt1 protein variants were expressed in BL21(DE3) (ThermoFisher Scientific, Waltham, MA, USA) (Figure 2). The pDS-pSer2 plasmid [20] was used as before [12] to incorporate pSer in response to a UAG codon at position 473 in ΔPHAkt1 and full-length Akt1 variants. To produce both full-length and PH domain–deficient Akt1 variants containing pSer473 (Supplementary Table S1), the pCDFDuet-1 Akt1–bearing plasmid was co-transformed with pDS-pSer2 into *Escherichia coli* Bl21(DE3) and plated on Luria broth (LB) agar plates with 25 μg/mL kanamycin and 50 μg/mL streptomycin. To produce pAkt1^T308^ variants, a pCDF-Duet1 plasmid containing both the Akt1 variant (MSC 2) and PDK1 (MSC 1) was transformed into *E. coli* BL21(DE3) and plated on LB agar plates with 50 μg/mL streptomycin. To produce ppAkt1^T308,S473^ variants, a pCDF-Duet1 plasmid containing both the Akt1 variant, MSC 2, with a TAG codon at position 473 and PDK1 variant, MSC 1 [12] was co-transformed with pDS-pSer2 into *E. coli* Bl21(DE3) and plated on LB agar plates with 25 μg/mL kanamycin and 50 μg/mL streptomycin. 

In all cases, a single colony was used to inoculate 70 mL of LB (with 50 μg/mL streptomycin and, if needed, 25 μg/mL kanamycin), which was grown, shaking, overnight at 37 °C. From this starter culture, a 10 mL inoculum was added to 1 L of LB with antibiotics (as above) and, for pSer473-containing variants only, *O*-phospho-l-serine (Sigma Aldrich, Oakville, ON, Canada) was added to a final concentration of 2.5 mM. The cultures were grown at 37 °C until OD_600_ = 0.6, at which point, for pSer473-containing variants only, 2.5 mM of additional pSer was added to the culture. Protein expression was induced by adding 300 µM of IPTG at OD_600_ = 0.8. Cultures were then incubated at 16 °C for 18 h. Cells were grown and pelleted at 5000× *g* and stored at −80 °C until further analysis. Akt1 protein variants were purified from the cell pellets using Ni-nitrilotriacetic acid affinity column chromatography (see Supplementary Material, affinity column chromatography description).

### 2.3. Parallel-Reaction Monitoring Mass Spectrometry of ppAkt1 

The ppAkt1 protein produced as described above and the commercially available active Akt1 (Abcam, lot 1, Cambridge, MA, USA) were precipitated in ice-cold acetone/ethanol/acetic acid (50/50/0.1, vol/vol/vol). The protein precipitate was resuspended in 8 M urea, then reduced in 5 mM dithiothreitol (DTT) at 37 °C for 1 h and alkylated in 14 mM iodoacetamide (IAA) in darkness at room temperature for 1 h. Unreacted IAA was neutralized by adding 5 mM DTT. The final protein concentration was determined by Bradford assay. Glu-C digestion was performed at 37 °C overnight with a Glu-C–to–protein ratio of 1:20 (*w*/*w*). The digest was desalted in C18 column (Phenomenex, Torance, CA, USA) according to the manufacturer’s protocol and resuspended in mass spectrometry (MS)-grade water. A Q Exactive Hybrid Quadrupole Orbitrap MS (Thermo Fisher Scientific, Waltham, MA, USA) was used to analyze the peptides. Data were analyzed using Skyline software [23].

### 2.4. MALDI-TOF/TOF Mass Spectrometry Analysis 

In-gel digestion was performed using MassPREP automated digester station (PerkinElmer, Downers Grove, IL, USA). Gel pieces were de-stained using 50 mM ammonium bicarbonate and 50% acetonitrile, which was followed by protein reduction using 10 mM DTT, alkylation using 55 mM IAA, and tryptic digestion in 50 mM ammonium bicarbonate, pH 8. Peptides were extracted using a solution of 1% formic acid and 2% acetonitrile and lyophilized. Prior to mass spectrometry analysis, dried peptide samples were re-dissolved in a 10% acetonitrile and 0.1% trifluoroacetic acid solution. MALDI matrix, a–cyano–4–hydroxycinnamic acid, was prepared as 5 mg/mL in 6 mM ammonium phosphate monobasic, 50% acetonitrile, 0.1% trifluoroacetic acid and mixed with the sample at a 1:1 ratio (*v*/*v*). Mass spectrometry data (Figure S1) were obtained using an AB Sciex 5800 MALDI TOF/TOF system (Framingham, MA, USA). Data acquisition and data processing were done using a TOF/TOF Series Explorer a nd Data Explorer (both from AB Sciex, Boston, MA, USA), respectively. The instrument was equipped with a 349 nm Nd:YLF OptiBeam On-Axis laser. The laser pulse rate was 400 Hz. Reflectron positive mode was used. Reflectron mode was externally calibrated at 50 ppm mass tolerance and internally at 10 ppm. Each mass spectrum was collected as a sum of 500 shots.

### 2.5. Akt1 Kinase Activity Assay

The activity of each Akt1 variant was characterized by performing kinase assays in the presence of 200 µM substrate peptide CKRPRAASFAE (SignalChem, Vancouver, BC, Canada) derived from the natural Akt1 substrate, glycogen synthase kinase (GSK-3β). Assays were performed in 3-(*N*-morpholino) propanesulfonic acid (MOPS, 25 mM, pH 7.0), β-glycerolphosphate (12.5 mM), MgCl_2_ (25 mM), ethylene glycol-bis(β-aminoethyl ether)-*N*,*N*,*N*′,*N*′-tetraacetic acid (EGTA, 5 mM, pH 8.0), ethylenediaminetetraacetic acid (EDTA) (2 mM), ATP (0.02 mM), and 0.4 µCi (0.033 μM) γ-[32P]-ATP in a 30 µL reaction volume. Reactions were incubated at 37 °C and time points were taken over 30 min time courses. As previously [12], reactions were initiated by the addition of 18 pmol of the indicated Akt1 variant to yield a final enzyme concentration of 600 nM and quenched by spotting on P81 paper [24]. For highly active Akt1 variants, the level of Akt1 was titrated to identify a linear range to accurately determine initial velocity (*v*_o_) (Figure S2). For this reason, Akt1 activity was compared based on the apparent catalytic rate (*k*_app_) = *v*_o_/[Akt1]. Samples from each reaction (5 µL) were spotted on P81 paper at specified time points. Following washes with 1% phosphoric acid (3 × 10 min) and 95% ethanol (1 × 5 min), the P81 paper was air-dried. Incorporation of ^32^P into the substrate peptide was detected by exposing the P81 paper to a phosphor-imaging screen. The ^32^P-peptide products were imaged and quantitated using a Storm 860 Molecular Imager and ImageQuant TL software (GE Healthcare, Mississauga, ON, Canada).

### 2.6. Kinase Inhibition Assay

A concentration gradient (0.001, 0.05, 0.5, 1, 5, 10 µM) of Akti-1/2 inhibitor VIII (Sellekchem, Houston, TX, USA) [25] was selected to determine the concentration dependence of Akt1 inhibition. Kinase inhibition assays were performed exactly as described above (Section 2.5) and with the addition of dimethyl sulfoxide at 10% (vol/vol) (control) or the indicated concentration of Akti-1/2 inhibitor. For each condition, initial velocity was determined over a time course of 10 min with time points at 0, 2, 5, and 10 min (Figure S3). In the inhibition assays, following established protocols [18], the inhibitor was preincubated with the enzyme for 5 min at 37 °C before addition of ATP and Akt1 substrate peptide to start the reaction. The time courses were used to determine the initial velocity of each reaction. The fraction of enzyme inhibition was measured based on initial velocities of uninhibited and inhibited reactions. From these data, half maximal inhibitory concentration (IC_50_) values were calculated using Sigma Plot (Systat Software, Inc., San Jose, CA, USA).

## 3. Results

### 3.1. Production of Recombinant Akt1 Variants 

We recently established a method to produce full-length Akt1 variants with programmed phosphorylations [12]. Here we applied this method to produced site-specifically phosphorylated Akt1 variants lacking the auto-inhibitory PH domain. The approach combines in vivo enzymatic phosphorylation with genetic code expansion to produce Akt1 variants containing either or both pThr308 and pSer473 (Figure 2). 

**Figure 3:**
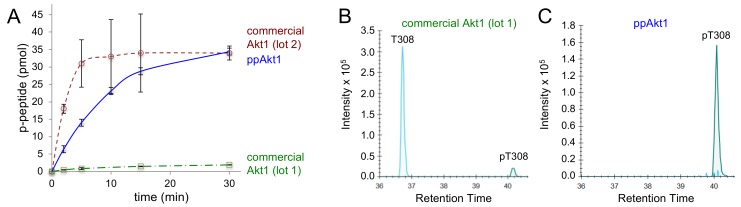
Activity of full-length ppAkt1 and commercially available active Akt1. (**A**) Kinase activity assays over a 30 min time course show substantially reduced activity of commercial Akt1 (lot 1, green squares) compared to full-length ppAkt1 (blue diamonds) produced in *E. coli*. Commercial Akt1 lot 2 (red circles) showed highly variable but similar activity to full-length ppAkt1. (**B**) Tryptic peptides from commercial Akt1 and ppAkt1 were analyzed by parallel-reaction monitoring mass spectrometry (PRM-MS). The purchased active Akt1 (lot 1) showed a low-intensity peak for phosphorylation at Thr308 (green peak at a retention time of ~40 min) and high-intensity peak for non-phosphorylated Thr308 (cyan peak, retention time of ~37 min). (**C**) PRM-MS analysis of ppAkt1^T308,S473^ showed a high-intensity peak for phosphorylation at Thr308 (green peak at a retention time of 40 min) and the non-phosphorylated Thr308 was undetectable.

Using genetic code expansion [12,20], we incorporated pSer in response to a UAG stop codon at position 473 in the relevant Akt1 constructs. Thr308 was site-specifically phosphorylated by co-expression of the upstream kinase PDK1 in *E. coli*. We [12] and others [26] have demonstrated that PDK1 is strictly specific in phosphorylating only the 308 site in Akt1. We used these methods in isolation or in combination to produce the three physiologically relevant Akt1 variants pAkt1^S473^, pAkt1^T308^, and ppAkt1^T308,S473^ (Figure 2). We observed that the ΔPH-Akt1 variants were all more soluble and were produced at 1.5- to 7-fold greater yield per liter of *E. coli* culture than the corresponding full-length Akt1 variants (Table 1). As previously reported [12], we confirmed phosphorylation of the Thr308 site (Figure 3C) and the Ser473 site (Figure S1) by mass spectrometry.

### 3.2. Recombinant Akt1 Produced in *E. coli* Versus Sf9 Cells 

Earlier work established production of partially active and truncated Akt1 in *E. coli*, which was unsuccessful at producing a sufficient amount of full-length Akt1 to determine activity [27]. Although we recently overcame these difficulties and developed a robust protocol to produce full-length Akt1 from *E. coli* with programmed phosphorylation, studies in the intervening period relied on insect Sf9 cell culture to produce recombinant Akt1 [28]. The ability to generate ppAkt1 from insect cells, however, requires a complex and low-yield in vitro procedure to phosphorylate Akt with two additional purified upstream kinases in the presence of lipid vesicles [28]. Protein production in Sf9 cells failed to yeild Akt1 with site-specific or programmed phosphorylation. The resulting protein was a mixture of singly and doubly phosphorylated species [29].

In order to benchmark the activity of the recombinant phosphorylated Akt1 protein we produced in *E. coli*, we compared the activity of full-length ppAkt1 to active Akt1 purchased from Abcam. The commercially available full-length Akt1 is made by a protocol similar to that established previously [28], in which Akt1 protein production in Sf9 cells is followed by in vitro phosphorylation of the purified Akt1 with purified PDK1. In testing an initial lot of active Akt1 (lot 1), we found that the commercially available Akt1 enzyme was catalytically deficient by eight-fold (Table 2) compared to the full-length ppAkt1 we produced (Figure 3A). 

Parallel-reaction monitoring mass spectrometry (PRM-MS/MS) was used to determine the identity of the residue at position 308 and the level of phosphorylation in ppAkt1 we produced and in the purchased protein. Mass spectrometry revealed that the low activity of lot 1 enzyme was attributable to the low level of phosphorylation at position 308 (peak intensity 1.3 × 10^1^) and a relatively high level of unphosphorylated Thr at position 308 (Figure 3B). In contrast, ppAkt1^T308,S473^ that we produced showed a high level (peak intensity 1.6 × 10^6^) of Thr308 phosphorylation and unphosphorylated Thr308 was not detected (Figure 3C). We then purchased a second lot of Akt1 enzyme (lot 2), and this enzyme displayed significantly more but highly variable activity compared to lot 1 (Table 2). Presumably, the second lot was quantitatively phosphorylated during production. In contrast to the method we developed for pAkt1 production in *E. coli* (Figure 2), the protocol to generate active Akt1 relying on Sf9 cells and subsequent in vitro phosphorylation appears to lead to a variable level of phosphorylation and activity in the resulting Akt1 preparations. 

### 3.3. Impact of PH Domain Deletion on Differentially Phosphorylated Akt1 

In order to determine the impact of the PH domain on each phospho-form of Akt1, we next assayed the activity of specifically phosphorylated ΔPH-Akt1 variants using γ-[32P]-ATP and a substrate peptide for Akt1 (CKRPRAASFAE) that was derived from GSK-3β, a well-established Akt1 substrate [12,30]. In kinase assay conditions that we previously optimized [12] to measure a wide range of Akt1 activity, we determined the apparent catalytic rate of each ΔPH-Akt1 variant (unphosphorylated, pAkt1^S473^, pAkt1^T308^, ppAkt1^T308,S473^). We then compared the apparent catalytic rates to the measurements we made previously with full-length Akt1 and pAkt1 variants to ascertain the relative impact of each phosphorylation state on auto-inhibition by the PH domain. 

**Figure 4:**
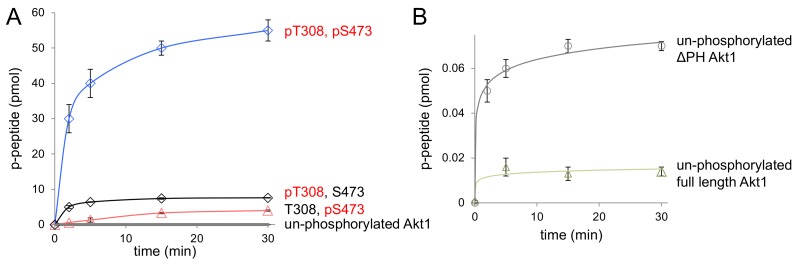
Enzyme activity of ΔPH-Akt1 variants. (**A**) The activity of differentially phosphorylated ΔPH Akt1 variants with the GSK-3β substrate peptide was measured over a 30 min time course. Akt1 phosphorylated at 308 and 473 (ΔPH-ppAkt^S473,T308^, blue diamonds) showed maximal activity compared to unphosphorylated ΔPH-Akt1 (gray circles) and singly phosphorylated Akt1 variants ΔPH-pAkt1^T308^ (black diamonds) and ΔPH-pAkt1^S473^ (pink triangles). (**B**) The basal activity of unphosphorylated ΔPH-Akt1 (gray circles) was compared to full-length unphosphorylated Akt1 (green triangles). All reported values represent the mean of triplicate experiments, with error bars indicating one standard deviation.

As anticipated, all Akt1 variants lacking the PH domain were significantly more active than their full-length counterparts (Table 2). Doubly phosphorylated ΔPH-Akt1 (ppAkt^T308,S473^) showed the highest activity among all three variants (Figure 4). The unphosphorylated ΔPH-Akt1 showed a basal level of activity that was significantly higher (2.7 ± 0.3-fold) than the background activity we recorded for full-length unphosphorylated Akt1 (Table 3). Although both unphosphorylated Akt1s would not have sufficient activity to induce Akt1-dependent signaling in cells [2,12], it is interesting to note that the auto-inhibitory effect of the PH domain is indeed measurable in the increased minimal activity of unphosphorylated ΔPH-Akt1 (Figure 4B).

Compared to unphosphorylated ΔPH-Akt1, the most active doubly phosphorylated Akt1 variant (ΔPH-ppAkt^T308,S473^) showed a 150,000-fold increase in apparent catalytic rate (Table 2, Figure 4). Since rapid enzyme kinetics were observed with 18 pmol of enzyme with both of these variants containing Thr308 phosphorylation, the enzyme concentrations were subsequently reduced by 10-fold and 100-fold to obtain a highly accurate initial velocity with which to determine the apparent rate (k_app_ = v_o_/[enzyme]) (Figure S2).

**Figure 5:**
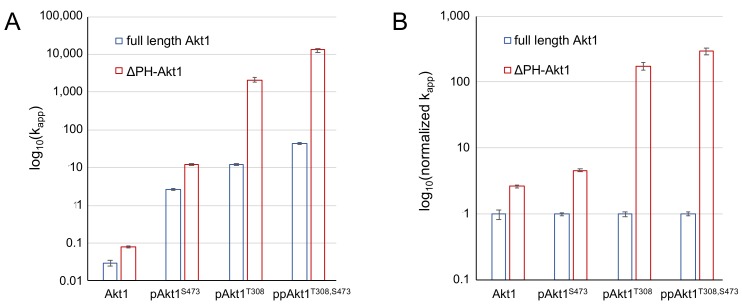
Impact of PH domain on the activity of differentially phosphorylated Akt1. (**A**) Apparent catalytic rates (*k*_app_) and (**B**) normalized *k*_app_ values of full-length Akt1 variants (blue) and ΔPH-Akt1 variants (red) are shown. Error bars represent one standard deviation of triplicate measurements.

Interestingly, dual phosphorylation of ΔPH-Akt1 led to a 100-fold greater increase in activity than that observed upon dual phosphorylation of the full-length enzyme, which was 1500-fold more active than the unphosphorylated full-length enzyme. The data indicate that the PH domain significantly dampens the catalytic activity endowed by phosphorylation at Thr308 and Ser473 (Figure 5). The ΔPH-Akt1 variant with a single phosphorylation at the Thr308 site was robustly active, 2500-fold above the unphosphorylated ΔPH-Akt1, yet with 60-fold reduced activity compared to the doubly phosphorylated enzyme ΔPH-ppAkt^T308/S473^ (Table 2). Phosphorylation at the C-terminal site Ser473 activated the enzyme 140-fold above the unphosphorylated control, but to a lesser extent (18-fold) than ΔPH-pAkt1^T308^. We previously observed a quantitatively similar pattern of activity with the full-length Akt1 variants (Table 2, [12]). 

We found that the PH domain exerts an auto-inhibitory effect, the strength of which depends on the phosphorylation status of the Akt1 enzyme. We compared apparent catalytic rates, *k*_app_, (Figure 5A) and normalized relative catalytic rates (Figure 5B) between the full-length [12] and PH domain–deficient Akt1 variants. In the context of a single phosphorylation at Ser473, the PH domain is associated with an approximately five-fold reduction in activity (Table 3, Figure 5). In the singly phosphorylated pAkt1^T308^ enzyme, the auto-inhibition was far stronger (175-fold). The two phosphorylations together led to a super-additive inhibitory effect (295-fold) in the doubly phosphorylated enzyme (Figure 5). 

### 3.4. Chemical Inhibition of Phosphorylated Akt1 Variants 

**Figure 6:**
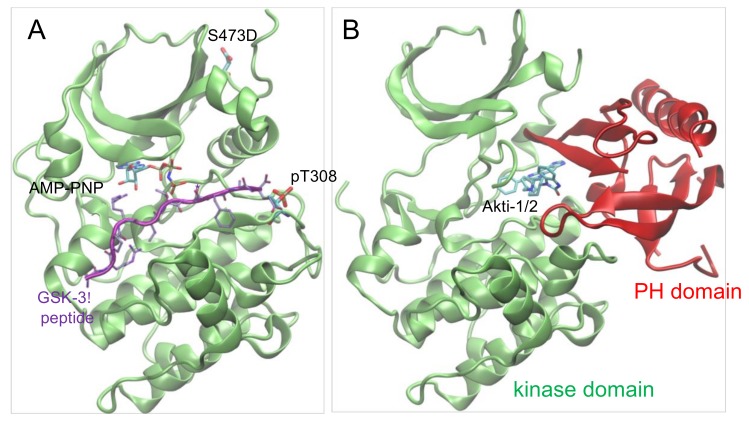
Structure of Akt1 in active and inhibitor bound forms. (**A**) Structure of ΔPH-pAkt1^Thr308^ Ser473Asp (PDB 1O6K [35]) is shown in complex with ATP analog (ANP-PNP) and substrate peptide (purple). (**B**) Structure of the full-length Akt1 (unphosphorylated) is shown in complex with the Akti-1/2 inhibitor VIII (PDB 1O96 [36]) binding in the cleft between the kinase domain (green) and the N-terminal PH domain (red).

Given the differential impact of Akt1 phosphorylation on auto-inhibition, we next identified phosphorylation dependence in the interaction between Akt1 and a clinically relevant drug scaffold, Akti-1/2 inhibitor VIII. Several classes of chemical inhibitors were developed to repress aberrant Akt1 activity in cancer cells [10]. Early Akt1 inhibitors focused on ATP competitive compounds [31], yet these molecules suffered from significant cross-reactivity with other AGC family kinases [32]. For this reason, subsequent efforts focused on allosteric Akt1 inhibitors. Akti-1/2 is an allosteric inhibitor that binds Akt1 in a cleft between the kinase and PH domains, locking the enzyme in a noncatalytic conformation (Figure 6). Biochemical data with different Akt1 preparations has provided estimates of IC_50_ of Akt1 for the Akti-1/2 inhibitor ranging from 58 nM to 2.7 μM [24,33,34] (Table 4). We suspected that this range of values resulted from preparations of Akt1 with various levels of phosphorylation, with less active Akt1 preparations leading to an underestimate of IC_50_. In addition, previous studies were unable to isolate the contribution of each regulatory phosphorylation site to inhibition by Akti-1/2.

**Figure 7:**
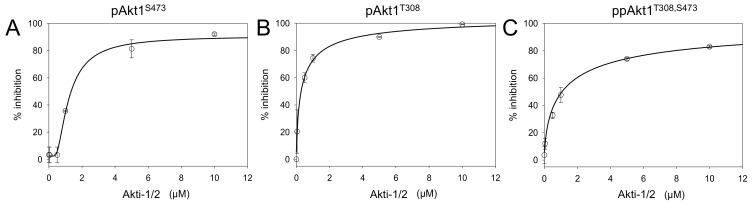
Chemical inhibition of full-length phosphorylated Akt1 variants. Inhibition of (**A**) pAkt1^S473^, (**B**) pAkt1^T308^, and (**C**) ppAkt1^T308,S473^ with varying concentrations (0.001, 0.05, 0.5, 1, 5, 10 μM) of Akti-1/2 inhibitor VIII. The resulting IC_50_ values are in Table 4.

We conducted Akt1 inhibition assays using Akti-1/2 concentrations varying from 0.01 to 10 μM. All three full-length phosphorylated Akt1 variants showed concentration-responsive enzyme inhibition (Figure 7, Figures S3–S6). Akti-1/2 was most potent (IC_50_ = 300 nM) when only the Thr308 site was phosphorylated (Table 4). Interestingly, at the lower concentrations of Akti-1/2 (0.001, 0.05, 0.5 μM), the compound was not effective at inhibiting the activity of pAkt1^S473^. Akt1 phosphorylated at the C-terminal Ser473 site was overall more resistant to inhibitor VIII inhibition, resulting in an IC_50_ value of 1.3 μM. In a surprising finding, the doubly phosphorylated enzyme had an indistinguishable IC_50_ from the pAkt1^S473^ enzyme. Together the data indicate that Ser473 phosphorylation provides a four-fold increase in resistance to the Akti-1/2 inhibitor even in the presence of phosphorylation at Thr308. This is the first report demonstrating phosphorylation dependence in the interaction between Akt1 and an inhibitor. 

## 4. Discussion

Akt1 is a prime target for therapeutic intervention due to its involvement in regulating a multitude of cellular pathways [37,38]. Aberrant Akt function has been linked to cancers and a variety of human diseases related to metabolic regulation, immune function, and neurological development [2]. The broad range of cellular processes governed by Akt, therefore, presents an opportunity for single-target therapeutic intervention in a variety of conditions. To capitalize on this opportunity, compounds that selectively attenuate Akt1 activity rather than global kinase inactivation are required. This need is reflected in clinical trials showing that nonspecific Akt inhibition in cancer therapy, due to inadvertent off-targeting of closely related kinases or multiple Akt isozymes, can result in detrimental side effects including liver damage and metabolic disorders [39,40,41,42]. Accordingly, the development of small molecule inhibitors for Akt reflects this sentiment of engineering greater target specificity [10]. From the earliest ATP-competitive inhibitors that unintentionally inhibited related AGC kinases, to more recent allosteric inhibitors that preferentially target Akt or even a subset of Akt isozymes, the continued pursuit for Akt1 inhibitor specificity requires an expanded understanding of the molecular basis of Akt1 activation and inhibition. 

Previous work was unable to uncover the role of each regulatory site (Thr308 and Ser473) in the inhibition kinetics of Akt1. Given this critical knowledge gap, we investigated the role of Akt1 phosphorylation status on the auto-inhibitory effect of the PH domain and on allosteric inhibition by Akti-1/2. 

### 4.1. Phosphorylation-Dependent Auto-Inhibition of Akt1

The N-terminal PH domain is shared among all three Akt isozymes and its direct function is to mediate protein–lipid interactions. It is well documented that PH domain binding to a lipid second messenger PIP_3_ in cell membranes releases the inhibitory effect of the PH domain [2]. Here, we produced differentially phosphorylated Akt1 variants with and without the PH domain in *E. coli* using genetic code expansion to incorporate pSer473, and the upstream Thr308 kinase PDK1 was co-expressed in *E. coli*, leading to quantitative phosphorylation of the recombinant Akt1 protein. Compared to unphosphorylated Akt1, all phosphorylated variants showed increased enzyme activity.

Previously, live cell imaging studies revealed that, once phosphorylated, Akt1 favors a PH-out conformation that increases Akt1 activity in the downstream phosphorylation of substrate proteins in both the cytoplasm and the nucleus. In addition, binding of Akt1 to PIP_3_ in the plasma membrane further releases the auto-inhibitory effect of the PH domain [43,44]. Although phosphorylation of the kinase domain at Thr308 is known to reduce the PH domain’s affinity for the kinase domain [42], the contribution of Thr308 and Ser473 phosphorylation to auto-inhibition by the PH domain was unknown. 

We quantitated the release of PH domain–mediated inhibition corresponding to each phosphorylation site and both sites in combination. Our data revealed that the magnitude of the auto-inhibitory effect of the PH domain was sensitively dependent on the phosphorylation status of Akt1. In comparison to the activity of full-length Akt1 phospho-variants, deletion of the PH domain significantly increased activity. In comparison to singly phosphorylated Akt1 variants, the auto-inhibitory effect of the PH domain was strongest (295-fold) in the most active and doubly phosphorylated Akt1 variant. Interestingly, the doubly phosphorylated Akt1 displayed increased activity upon PH domain deletion that was greater than the sum of the effects observed for the singly phosphorylated Akt1 variants. The data suggest a novel finding that the PH domain dampens the activity of Akt1 variants in a phosphorylation-dependent manner.

The combination of phosphorylation and PH domain release regulates the kinase activity of Akt1 over a 400,000-fold range. The data indicate the degree to which the unphosphorylated PH-in conformation of Akt1 is suppressed in comparison to the PH-out doubly phosphorylated version of the enzyme that exists at the plasma membrane in living cells. Recent in vitro biochemical work suggested that association with PIP_3_-containing lipid vesicles stimulated the activity of a full-length Akt1 protein by seven-fold [44]. The authors suggested that this may be an underestimate due to low levels of phosphorylation (10% pThr308, 1% pSer473) in their Akt1 prepared from Sf9 cells. Our data suggest that release of the PH domain in the context of doubly phosphorylated Akt1 would lead to a maximal activity increase of 300-fold; a PH domain–dependent reduction lower than this value would indicate that there may be significant interaction between the Akt1 kinase and PH domains even in the PIP_3_-bound state at the plasma membrane. 

Indeed, a recent debate in the literature [45] is under way to explain how Akt1 activity can be maintained far from the membrane. Recent findings suggest that Akt1 is dephosphorylated on a time scale of ~10 min following dissociation from PIP_3_ on the plasma membrane [46]. Multiple nuclear substrates of Akt1 are, however, known to be phosphorylated much more rapidly, on a time scale of seconds [47]. Although the authors suggested that all or perhaps most of the active Akt1 is membrane-associated [46], this finding disputes several independent studies identifying many cytosolic and nuclear targets of Akt1-dependent phosphorylation. Multiple studies clearly identified both phospho-Akt1 (e.g., [47,48,49]) and directly measured Akt1 activity in the nucleus [17]. Using a fluorescent reporter that quantifies Akt1 activity in live cells in real time, Kunkel and Newton observed Akt1 activity distributed in both the cytoplasm and nucleus on a time scale of seconds to minutes following growth factor stimulation. Their data further show similar levels of Akt1 activity in the nucleus and cytoplasm, with a delay in the peak activity in the nucleus of ~2 min. Additional experiments confirmed that this lag is related to the time it takes for active Akt1 to translocate from the plasma membrane to the nucleus [17]. In light of our data and these findings, membrane-associated Akt1 is likely significantly more active than doubly phosphorylated Akt1 found in the nucleus and cytosol. Phosphorylated Akt1, nevertheless, does persist away from the membrane, retaining significant activity in comparison to unphosphorylated Akt1.

### 4.2. Phosphorylation-Dependent Chemical Inhibition of Akt1

Posttranslational modifications, of which phosphorylation is the most common, are ubiquitous mechanisms that cells use to tune the activity of individual proteins and increase the functional diversity of the proteome. Phosphorylation can have a significant impact on the activity of proteins (kinases are a prime example), and the phosphorylation status of a protein target can accordingly impact the ability of small molecules to inhibit enzyme activity. In the experiments presented here, we determined precisely how the phosphorylation status of Akt1 influences the repression of kinase activity by chemical inhibition. We investigated the Akt-specific allosteric inhibitor Akti-1/2. Based on the results of our assays, the phosphorylation status of Akt1 can indeed influence the degree to which Akti-1/2 is able to act on Akt1. Akti-1/2 was most effective at inhibiting pAkt1^T308^, whereas phosphorylation at position Ser473 in either pAkt^S473^ or ppAkt^T308/S473^ reduced the relative effectiveness of the inhibitor by four-fold. 

Ser473 phosphorylation appears to act as a built-in mechanism of drug resistance for Akt1. It is well established that hyperphosphorylation of Akt1 at both regulatory sites is linked to poor prognosis and therapeutic resistance [3,4,5,6,50]. The accepted model for Akti-1/2 activity suggests that the compound locks Akt1 into the auto-inhibited or PH-in conformation and prevents Akt1 from binding PIP_3_ in the plasma membrane, inhibiting phosphorylation at Thr308 and Ser473 [51]. Our data also suggest that, although limited by Ser473 phosphorylation, Akti-1/2 has significant activity in inhibiting the pre-phosphorylated Akt1 variants that the inhibitor initially encounters in the cell. Studies in cell culture and mouse models show that the short-term impact of Akti-1/2 treatment is that pThr308 and pSer473 levels are depleted within hours. A related allosteric Akt inhibitor, and the most clinically promising [52] compound, MK-2206 significantly reduces Ser473 phosphorylation after 10 h in mouse models with patient-derived xenografts of endometrial tumors [53]. 

Surprisingly, breast cancer cells (BT474) treated with Akti-1/2 (1 μM) showed a rebound effect in Akt1 phosphorylation status following Akti-1/2 treatment. These cells have amplified *HER2* genes and constitutively activate the PI3K/Akt signaling cascade. In short-term experiments (minutes to hours), Akt1 phosphorylation reduced in response to inhibitor treatment, but at longer times (2–3 days) Akt1 phosphorylation status returned to stimulated levels in these cancer cells. In this longer time frame, phosphorylation of the downstream Akt1 target S6K was not restored, but PRAS40 phosphorylation was partially restored concomitant with increased Akt1 phospho-status. The study provided evidence that Akti-1/2 ultimately induces the expression and activation of receptor tyrosine kinases EGFR, HER3, and HER4, which in turn reactivate Akt1 [54]. Interestingly, in mouse models a similar rebound in Ser473 phosphorylation following treatment with MK-2206 was observed on time courses of 5 to 20 days [55]. In these cases, Ser473 phosphorylation may provide the tumor cell with a means to reduce the effectiveness of Akt1 inhibitors. The data suggest a need to develop an inhibitor that is more effective against the most active and doubly phosphorylated form of Akt1.

### 4.3. Synthetic Biology Approach to Generate Active Akt1 

In our current study and previous work [12,19,20,21], we demonstrated that genetic code expansion with pSer in *E. coli* provides a simple route to produce designer phosphoproteins. Here, we produced differentially phosphorylated Akt1 variants with and without the PH domain in *E. coli* using genetic code expansion and enzymatic phosphorylation in *E. coli*. We found that the variants lacking the PH domain were produced at significantly higher yield compared to the full-length Akt1 protein. The ability to produce recombinant Akt1 protein with programmed phosphorylation(s) was essential for our investigation into the role of each phospho-site in Akt1 inhibition. 

In agreement with our previous work [12], we found that the system we developed to produce Akt1 with either or both regulatory phosphorylation sites (Figure 2) leads to a consistently active Akt1 variant with indistinguishable batch-to-batch variability. This is in contrast to the commercially available Akt1 produced using the Sf9 cell system followed by in vitro PDK1 phosphorylation of Thr308 [28]. We observed vast batch-to-batch variability of enzyme activity in the commercially available Akt1, which warrants cautious attention in its experimental use. Previous work also established that Akt1 produced in Sf9 cells leads to a mixture of singly and doubly phosphorylated Akt1 variants [29], at times leading to low stoichiometry of phosphorylation [44] (Figure 3B). Our approach consistently results in site-specifically phosphorylated and active Akt1 variants that provide a reliable and consistent source of protein for biochemical studies and applications in inhibitor screening.

## Figures and Tables

**Table 1:** Protein yields for Akt1 variants.

	Protein Yields (μg/L *Escherichia coli* Culture)	
Akt1 Variant	Full Length	ΔPH Akt1
Akt1 (unphosphorylated)	46	330
pAkt1^S473^	100	150
pAkt1^T308^	37	235
ppAkt1^T308,S473^	45	224

**Table 2:** Activity of Akt1 variants.

Akt1 Variant	Akt1 Amount (pmol)	Initial Velocity *v*_o_ (fmol/min)	Apparent Catalytic Rate *k*_app_ (fmol/min/pmol Akt1)	Activation (Fold Increase)	Reference
Akt1 (unphosphorylated)	18	0.6 ± 0.2	0.03 ± 0.01	1.0 ± 0.3	[12]
pAkt1^S473^	18	46 ± 5	2.6 ± 0.3	85 ± 9	[12]
pAkt1^T308^	1.8	22 ± 4	12 ± 2	400 ± 70	[12]
ppAkt1^T308,S473^	1.8	79 ± 11	44 ± 6	1500 ± 200	[12]
ΔPHAkt1 (unphosphorylated)	18	1.4 ± 0.1	0.079 ± 0.006	2.7 ± 0.2	this study
ΔPH pAkt1^S473^	18	210 ± 20	12 ± 1	390 ± 40	this study
ΔPH pAkt1^T308^	0.18	370 ± 100	2100 ± 600	6900 ± 1800	this study
ΔPH ppAkt1^T308,S473^	0.18	2300 ± 600	(13 ± 3) × 10^3^	(4 ± 1) × 10^5^	this study
Commercial Akt1 (Abcam)					
Lot 1	18	100 ± 20	6 ± 1	200 ± 30	this study
Lot 2	18	2200 ± 700	120 ± 40	4000 ± 1000	this study

**Table 3:** Relative activity of full-length Akt1 versus variants lacking the PH domain (ΔPH Akt1) *.

Akt1 Variant	Full-Length Akt1	ΔPH-Akt1
Akt1 (unphosphorylated)	1.0 ± 0.3	2.7 ± 0.2
pAkt1^S473^	1.0 ± 0.1	4.6 ± 0.4
pAkt1^T308^	1.0 ± 0.2	175 ± 50
ppAkt1^T308,S473^	1.0 ± 0.1	295 ± 68

* The fold increase in *k*_app_ for each ΔPH-Akt1 enzyme variant was calculated by normalizing to the corresponding full-length Akt1 variant k_app_.

**Table 4:** Akti-1/2 inhibitor half maximal inhibitory concentration (IC_50_) for pAkt1 variants.

Akt1 Variant	Inhibitor Type	IC_50_ (μM)	Expression System	Reference
ppAkt1^T308,S473^	Akti-1/2 inhibitor VIII	1.2 ± 0.3	*E. coli*	this study
pAkt1^T308^	Akti-1/2 inhibitor VIII	0.3 ± 0.1	*E. coli*	this study
pAkt1^S473^	Akti-1/2 inhibitor VIII	1.3 ± 0.1	*E. coli*	this study
active	Akti-1/2 inhibitor VIII	0.058	*Drosophila* S2 cells	[25]
active	Akti-1/2 inhibitor VIII	0.1	HEK 293 cells	[33]
active	Akti-1/2 inhibitor	2.7	*Drosophila* S2 cells	[34]
active	Akti-1 inhibitor	4.6	*Drosophila* S2 cells	[34]

## Supplementary Materials

The following are available online at http://www.mdpi.com/2073-4425/9/9/450/s1, Supplementary Materials and Methods, Table S1: Plasmids used in this study, Figure S1: Mass spectra confirming genetically encoded phosphoserine in ΔPH-ppAkt1, Figure S2: Initial velocity measurements for highly active ΔPH-Akt1 variants, Figure S3: Inhibition of full-length Akt1 variants incubated with Akti-1/2 inhibitor VIII, Figure S4: Autoradiographs of Akt1 inhibitor assays with ppAkt1T308, S473, Figure S5: Autoradiographs of Akt1 inhibitor assays with pAkt1T308, Figure S6: Autoradiographs of Akt1 inhibitor assays with pAkt1S473.
