# Supplementary material for: Phosphorylation-Dependent Inhibition of Akt1
Source: Genes (Basel). 2018 Sep 7;9(9):450. doi: 10.3390/genes9090450 (PMC6162393; doi:10.3390/genes9090450)
Supplement: Supplementary file 1 [file genes-09-00450-s001.pdf]

Supplementary Materials for

Phosphorylation-dependent inhibition of Akt1

Nileeka Balasuriya[1,], McShane McKenna[1], Xuguang Liu[1], Shawn S. C. Li[1], and Patrick O'Donoghue[1,2,†]

[1]Department of Biochemistry, [2]Department of Chemistry, The University of Western Ontario, London, ON N6A 5C1, Canada.

†To whom correspondence should be addressed:
Email: patrick.odonoghue@uwo.ca

Supplementary Materials and Methods

Affinity column chromatography. The His-tagged proteins were purified using Ni affinity column chromatography. 0.5 ml of Ni-NTA resin (Thermo-Fisher Scientific) was used for 1 l of *E. coli* culture. The cell lysates (see Methods) were loaded into the column (2 ml column volume) and washed with 20 column volumes of wash buffer A (20 mM Hepes, 150 mM NaCl, 3 mM β-mercaptoethanol, 3mM Dithiothreitol, 1 mM Na_3VO_4 and 5 mM NaF, 15 mM imidazole) followed by 10 column volumes of wash buffer B (20 mM Hepes, 150 mM NaCl, 3 mM β-mercaptoethanol, 3mM DTT, 1 mM Na_3VO_4 and 5 mM NaF, 20 mM imidazole). Protein was eluted using an elution buffer (20 mM Hepes, 150 mM NaCl, 3 mM β-mercaptoethanol, 3mM dithiothreitol, 1 mM Na_3VO_4 and 5 mM NaF) with 75 mM Imidazole. The same protocol was used for Akt1 variants with phosphomimetic mutations except: 1) phosphatase inhibitors were excluded from the buffers, 2) the imidazole concentrations of the wash buffers were 20 - 50 mM, and 3) the imidazole concentration of the elution buffer was 200 mM. Fractions were run on 10% SDS (sodium dodecyl sulfate) poly-acrylamide gels. As detailed previously [1], Akt1 variants were further purified by size exclusion chromatography.

Supplementary References

1. Balasuriya, N.; Kunkel, M.T.; Liu, X.; Biggar, K.K.; Li, S.S.; Newton, A.C.; O'Donoghue, P. Genetic code expansion and live cell imaging reveal that thr308 phosphorylation is irreplaceable and sufficient for akt1 activity. *J Biol Chem* **2018**, *293*, 10744-10756.
2. George, S.; Aguirre, J.D.; Spratt, D.E.; Bi, Y.; Jeffery, M.; Shaw, G.S.; O'Donoghue, P. Generation of phospho-ubiquitin variants by orthogonal translation reveals codon skipping. *FEBS Lett* **2016**, *590*, 1530-1542.

Table S1. Plasmids used in this study.

Plasmid ID	Plasmid backbone	Selectable marker	Inserted genes	Genetic modifications	Protein product
(1) WT Akt1	pDS0 (pUC) [2]	Amp	*AKT1*	None.	Akt1, unphosphorylated. (full-length, inactive) [1]
(2) WT Akt1-PDK1	pCDF-Duet-1	Strep	*AKT1* *PDK1*	None.	Akt1, phosphorylated at position T308. (full-length, active) [1]
(3) Akt1 UAG473	pCDF-Duet-1	Strep	*AKT1*	UAG mutation at position 473 in *AKT1*.	Akt1, phosphorylated at position S473. (full-length, slightly active) [1]
(4) Akt1-UAG473-PDK1	pCDF-Duet-1	Strep	*AKT1* *PDK1*	UAG mutation at position 473 in *AKT1*.	Akt1, phosphorylated at positions T308 and S472. (full-length, highly active) [1]
(5) pSer-OTS	pDS-pSer2	Kan	tRNA^{sep} (5x) SepRS9 EFSep21	This system is comprised of the 2nd generation mutants of the pSer incorporation system.	pSer orthogonal translation system, capable of decoding UAG codons and incorporating pSer into nascent peptides [2]
(6) ΔPH-WT Akt1	pDS0 (pUC) [2]	Amp	*AKT1*	Deletion of the PH domain from *AKT1* (Δ residues 2-109).	ΔPH-Akt1, unphosphorylated. (truncated, inactive)
(7) ΔPH-WT Akt1-PDK1	pCDF-Duet-1	Strep	*AKT1* *PDK1*	Deletion of the PH domain from *AKT1* (Δ residues 2-109).	ΔPH-Akt1, phosphorylated at position T308. (truncated, active)
(8) ΔPH-Akt1-UAG473	pCDF-Duet-1	Strep	*AKT1*	Deletion of the PH domain from *AKT1* (Δ residues 2-109). UAG mutation at position 473 in *AKT1*	ΔPH-Akt1, phosphorylated at position S473. (truncated, slightly active)
(9) ΔPH-Akt1 UAG473-PDK1	pCDF-Duet-1	Strep	*AKT1* *PDK1*	Deletion of the PH domain from *AKT1* (Δ residues 2-109). UAG mutation at position 473 in *AKT1*.	ΔPH-Akt1, phosphorylated at positions T308 and S472. (truncated, highly active)

Supplementary Figures

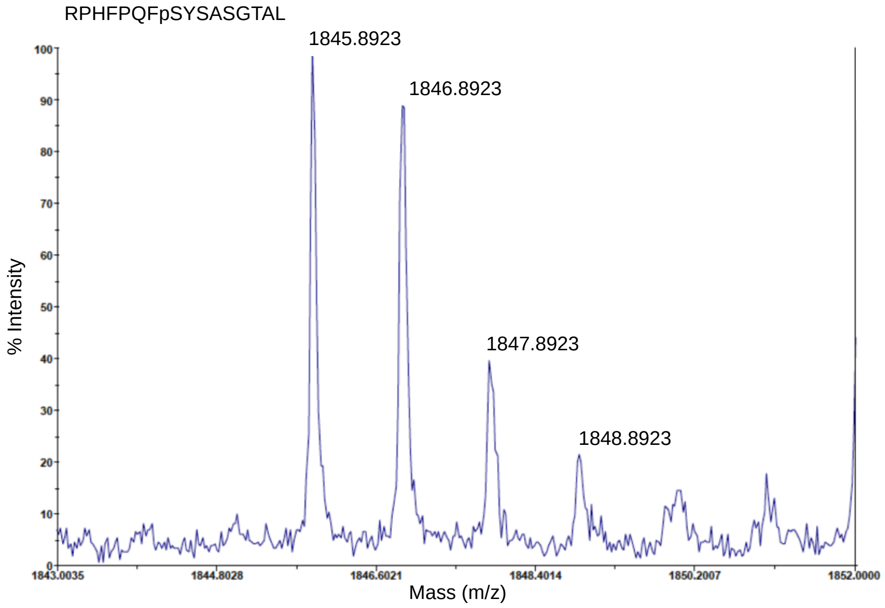

Figure S1. Mass spectra confirming genetically encoded phosphoserine in ΔPH-ppAkt1. Tryptic digested peptides of ΔPH-ppAkt1^{T308,S473} were subjected to MALDI-TOF mass spectrometry The C-terminal peptide of ΔPH-Akt1 carrying a single phosphorylation was readily detected. The isotopic masses of the peptide carrying the most abundant peak of ^{12}C isotope (observed m/z = 1845.8923; expected m/z = 1845.8378) and the 3 consecutive peaks with ^{13}C isotopes (m/z = 1846.8923, 1847.8923, 1848.8923, respectively) are shown.

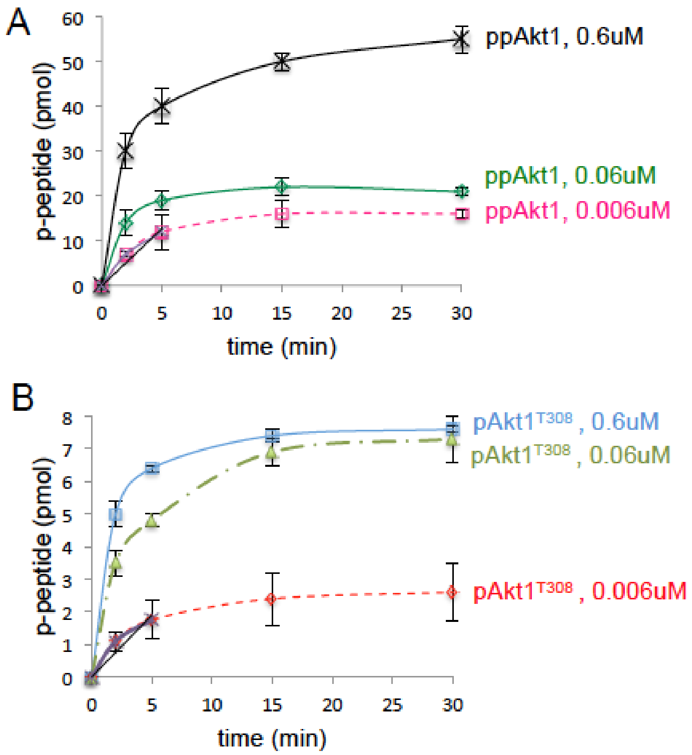

Figure S2. Initial velocity measurements for highly active ΔPH Akt1 variants. (**A**) ΔPH-ppAkt1$^{T308, S473}$ and (**B**) ΔPH-pAkt1^{T308} were diluted by 10-fold and 100-fold, respectively, to obtain a linear range of increasing enzyme activity. Initial velocities were calculated using the data points at the linear range of 100-fold diluted enzyme.

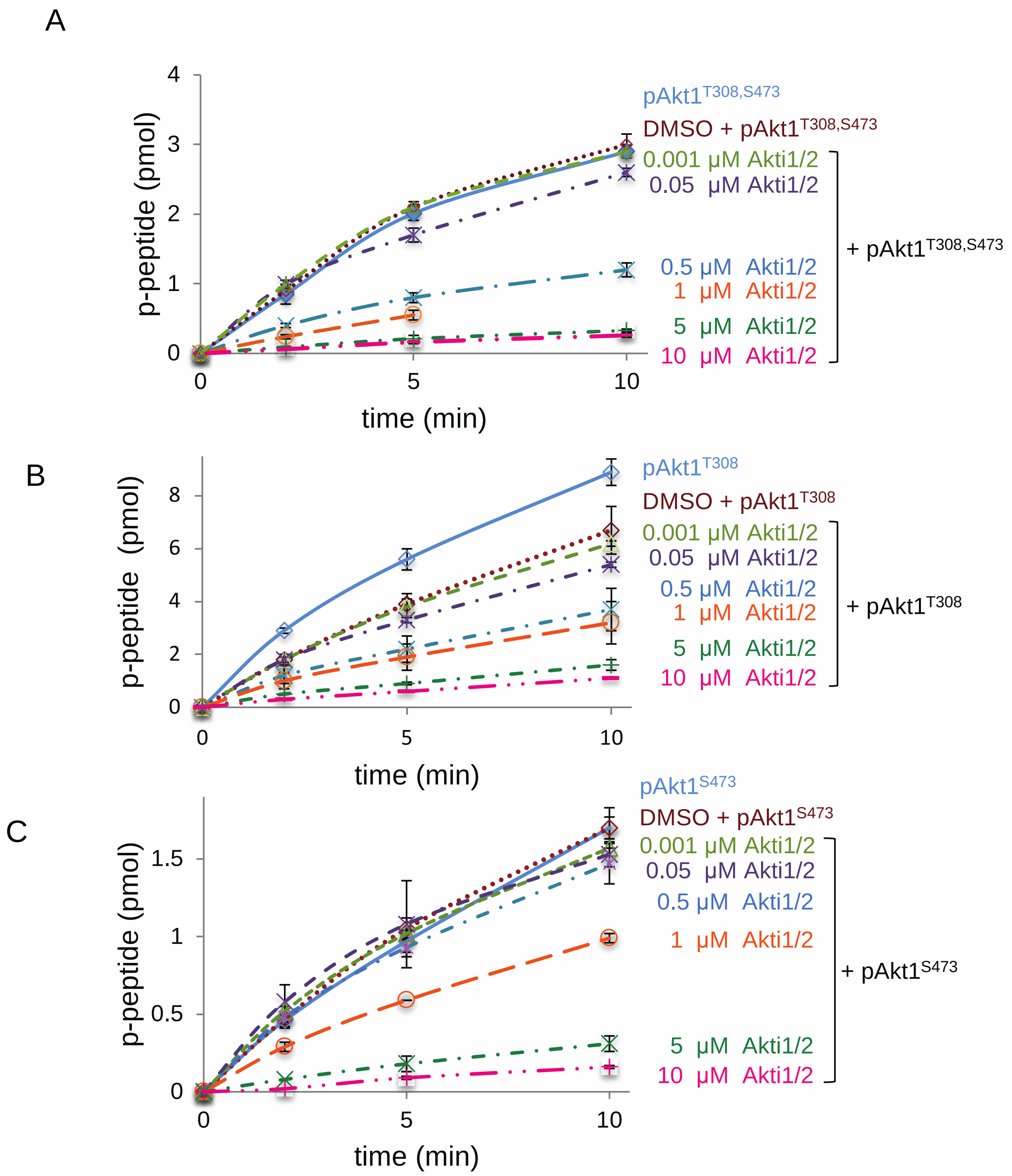

Figure S3. Inhibition of the full length Akt1 variants incubated with Akti-1/2 inhibitor VIII. (A) ppAkt1$^{T308, S473}$, (B) pAkt^{T308}, (3) pAkt1^{S473} variants were incubated with increasing concentrations (0.001 uM to 10 uM) of Akti1/2. Error bars indicate 1 standard deviation based on triplicate experiments.

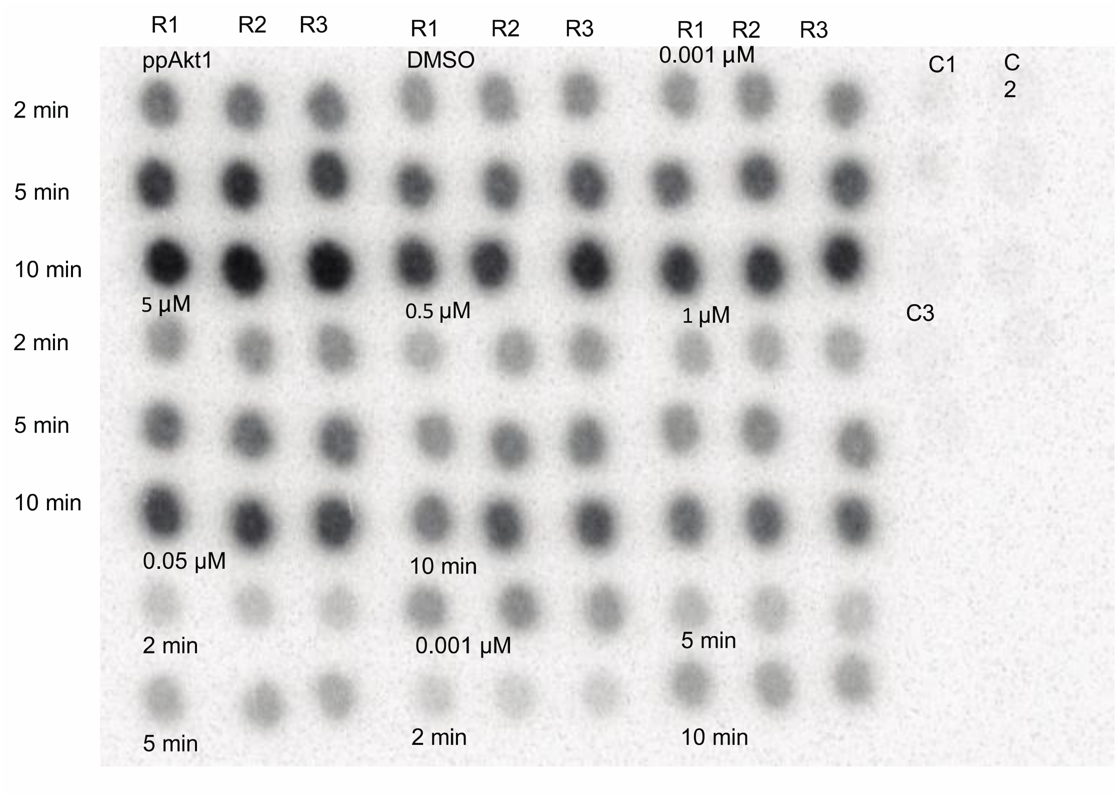

Figure S4. Autoradiographs of Akt1 inhibitor assays with ppAkt1$^{T308, S473}$. Time courses are shown for the reaction catalyzed by ppAkt1^{T308,S473} with the indicated concentrations of inhibitor Akti1/2. Assays were performed in triplicate (as indicated by R1-R3). Controls include: C1 without substrate peptide, C2 without enzyme, C3 only kinase assay buffer.

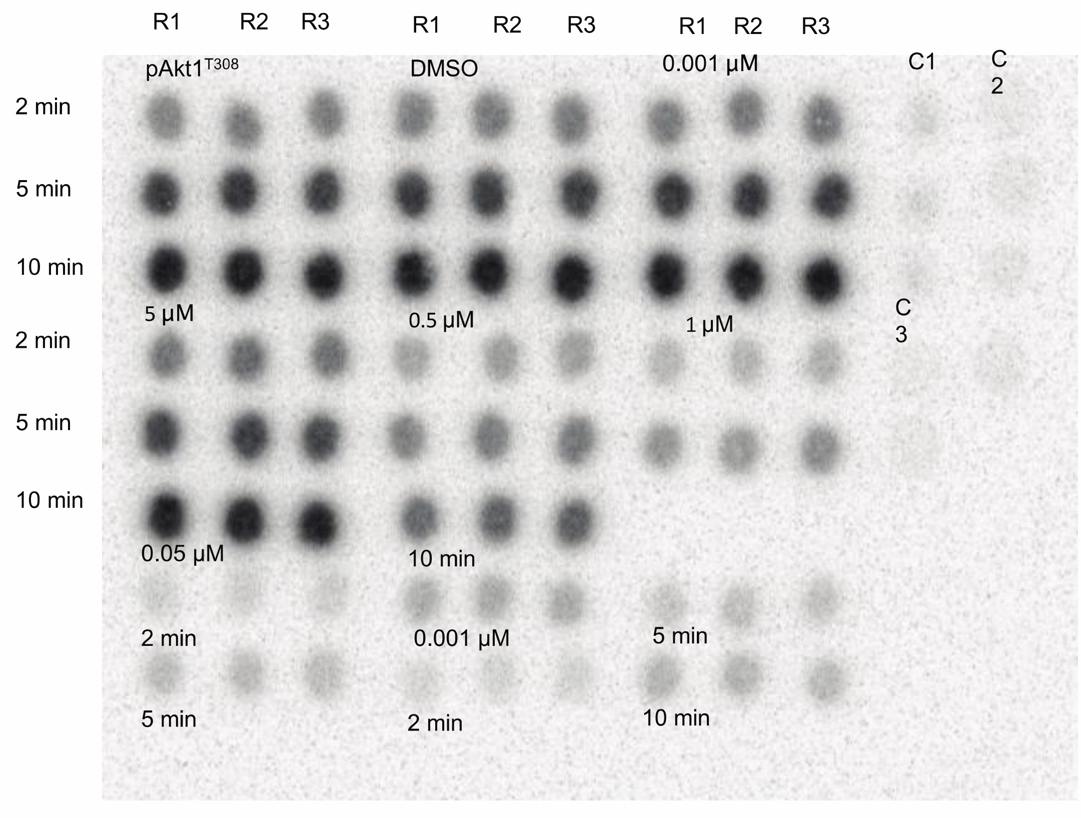

Figure S5. Autoradiographs of Akt1 inhibitor assays with pAkt1^{T308}. Time courses are shown for the reaction catalyzed by pAkt1^{T308} with the indicated concentrations of inhibitor Akti1/2. Assays were performed in triplicate (as indicated by R1-R3). Controls include: C1 without substrate peptide, C2 without enzyme, C3 only kinase assay buffer.

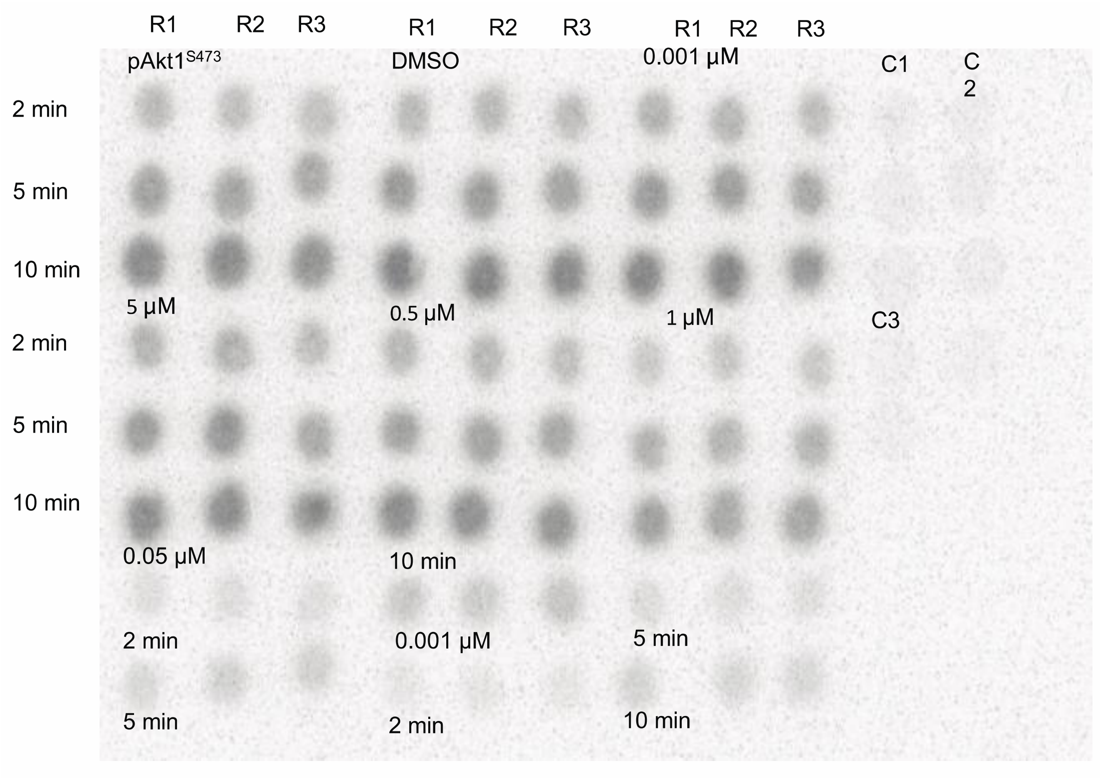

Figure S6. Autoradiographs of Akt1 inhibitor assays with pAkt1^{S473}. Time courses are shown for the reaction catalyzed by pAkt1^{S473} with the indicated concentrations of inhibitor Akti1/2. Assays were performed in triplicate (as indicated by R1-R3). Controls include: C1 without substrate peptide, C2 without enzyme, C3 only kinase assay buffer.
